# Biosynthesis of plant-specific stilbene polyketides in metabolically engineered *Escherichia coli*

**DOI:** 10.1186/1472-6750-6-22

**Published:** 2006-03-21

**Authors:** Kevin T Watts, Pyung C Lee, Claudia Schmidt-Dannert

**Affiliations:** 1 Department of Biochemistry, Molecular Biology and Biophysics, University of Minnesota, St. Paul, MN 55108, USA

## Abstract

**Background:**

Phenylpropanoids are the precursors to a range of important plant metabolites such as the cell wall constituent lignin and the secondary metabolites belonging to the flavonoid/stilbene class of compounds. The latter class of plant natural products has been shown to function in a wide range of biological activities. During the last few years an increasing number of health benefits have been associated with these compounds. In particular, they demonstrate potent antioxidant activity and the ability to selectively inhibit certain tyrosine kinases. Biosynthesis of many medicinally important plant secondary metabolites, including stilbenes, is frequently not very well understood and under tight spatial and temporal control, limiting their availability from plant sources. As an alternative, we sought to develop an approach for the biosynthesis of diverse stilbenes by engineered recombinant microbial cells.

**Results:**

A pathway for stilbene biosynthesis was constructed in *Escherichia coli *with 4-coumaroyl CoA ligase 1 4CL1) from *Arabidopsis thaliana *and stilbene synthase (STS) cloned from *Arachis hypogaea*. *E. coli *cultures expressing these enzymes together converted the phenylpropionic acid precursor 4-coumaric acid, added to the growth medium, to the stilbene resveratrol (>100 mg/L). Caffeic acid, added in the same way, resulted in the production of the expected dihydroxylated stilbene, piceatannol (>10 mg/L). Ferulic acid, however, was not converted to the expected stilbene product, isorhapontigenin. Substitution of 4CL1 with a homologous enzyme, 4CL4, with a preference for ferulic acid over 4-coumaric acid, had no effect on the conversion of ferulic acid. Accumulation of tri- and tetraketide lactones from ferulic acid, regardless of the CoA-ligase expressed in *E. coli*, suggests that STS cannot properly accommodate and fold the tetraketide intermediate to the corresponding stilbene structure.

**Conclusion:**

Phenylpropionic acids, such as 4-coumaric acid and caffeic acid, can be efficiently converted to stilbene compounds by recombinant *E. coli *cells expressing plant biosynthetic genes. Optimization of precursor conversion and cyclization of the bulky ferulic acid precursor by host metabolic engineering and protein engineering may afford the synthesis of even more structurally diverse stilbene compounds.

## Background

Flavonoids and stilbenes are plant-specific natural products with a wide range of biological activities, such as UV protection, as signals of bacterial root nodulation, coloration and defense against herbivory and microbial pathogens[1,2]. The uncovering of an increasing number of health benefits associated with these compounds has resulted in an explosion of research on their medicinal properties during the last few years[3,4]. In particular, the stilbene compound resveratrol has received considerable attention for its potential medicinal properties [5-7], and has been identified as a compound promoting increased lifespan through activation of sirtuin deacetylases[8], although the exact effect of resveratrol on Sir2 family proteins remains controversial[9,10]. Less controversial is the effect of stilbenes on protein-tyrosine kinases, with piceatannol having been shown as a selective inhibitor of the human tyrosine kinase Syk[11].

**Figure 1:**
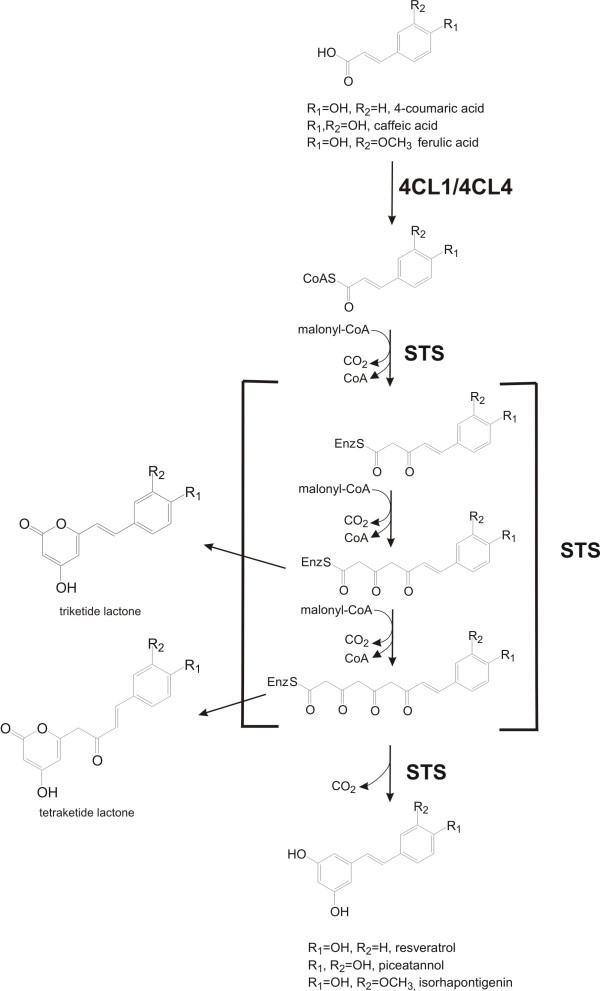
**Engineered route to stilbene biosynthesis in *E. coli***. Stilbene biosynthesis takes place by sequential addition of acetate units, derived from the decarboxylation of malonyl-CoA, to a CoA activated phenylpropionic acid. Polyketide intermediates (in brackets), if not cyclized properly, can be released from stilbene synthase (STS) and spontaneously form lactone derailment products. Acceptable starter units are extended to a linear tetraketide intermediate, which then undergoes aldol condensation and decarboxylation, followed by aromatization, to produce the proper stilbene structure. *E. coli *was engineered to produce stilbenes through the biotransformation of phenylpropionic acids by the enzymes 4-coumaroyl:CoA ligase 1 (4CL1) or 4-coumaroyl:CoA ligase 4 (4CL4) and STS. 4-coumaric acid and caffeic acid were transformed to the expected stilbene compounds, resveratrol and piceatannol, using this pathway. Ferulic acid was not converted to the corresponding stilbene using either 4CL1 or 4CL4, but lactone derailment products were detected, indicating that this substrate can be used as a starter unit *in vivo*.

Flavonoids and stilbenes are synthesized from a coenzyme A (CoA) activated phenylpropanoid starter unit and three malonyl-CoA extender units (Fig. 1). The first step in phenylpropanoid biosynthesis is the deamination of L-phenylalanine to *trans*-cinnamic acid, catalyzed by phenylalanine ammonia lyase (PAL) (EC 4.3.1.5). Cinnamic acid is hydroxylated by cinnamate-4-hydroxylase (C4H) (EC 1.14.13.11) to make 4-coumaric acid, which is then activated by 4-coumaroyl:CoA ligase (4CL) (EC 6.2.1.12) to make 4-coumaroyl-CoA. A type III polyketide synthase then sequentially adds three acetate extender units, derived from malonyl-CoA, to a single activated 4-coumaroyl-CoA starter unit. Depending on the polyketide synthase activity, chalcone synthase (CHS, EC 2.3.1.74) or stilbene synthase (STS, EC 2.3.1.95), subsequent folding and cyclization of the generated tetraketide intermediate results either in the production of a chalcone or stilbene ring structure.

Expression of plant secondary metabolic pathways, including those for flavonoid and stilbene biosynthesis, are typically under tight temporal and spatial control [12-14], which limits the availability of many medicinally important plant natural products. As an alternative biosynthetic host, microbial cells may be engineered for the production of plant derived natural products. In an attempt to access plant-derived flavonoid compounds in engineered microbial cells, we have previously shown that the *Arabidopsis thaliana *flavonoid biosynthetic pathway can be functionally assembled in recombinant *E. coli *for the biosynthesis of flavonoids[15]. Here we describe the cloning of a stilbene synthase from *Arachis hypogaea *(peanut) and its functional co-expression with two 4CL enzymes for the biotransformation of phenylpropionic acid precursors to modified stilbene compounds in *E. coli*. Biotransformation of structurally diverse phenylpropionic acids using recombinant *E. coli *opens up the possibility to produce functionalized stilbene compounds without the need of additional biosynthetic enzymes that may be difficult to express functionally in *E. coli*, such as plant cytochrome P450 monooxygenases. We observe for the first time production of two stilbene compounds by *E. coli*, with the stilbene resveratrol produced at a level of over 100 mg/L.

## Results and discussion

### Cloning and expression of *A. hypogaea *stilbene synthase (STS)

Stilbene synthases have been characterized from several plant species, including *Pinus sylvestris *and *A. hypogaea*, both of which have structural data reported[16,17]. For the purpose of synthesizing structurally diverse stilbene compounds in *E. coli*, we chose to use the STS from *A. hypogaea *because of its reported broad substrate specificity[18,19]. Peanut seeds were purchased from a commercial supplier and grown for approximately two weeks before preparation of cDNA. Two preparations of cDNA were made, one from fully opened leaves and one from a combination of roots and root hairs together. The cDNA's were probed with gene specific [Genbank: AB027606] primers, and a PCR product of the expected size (approx. 1200 bp) was only obtained from root cDNA, whereas the leaf cDNA gave no detectable amplification product [see Additional file 1]. The root cDNA PCR product was then cloned into the expression vector pUCMod. This plasmid was constructed by deletion of the pUC19 operator sequence, which results in constitutive expression from the *lac *promoter[20]. The sequence obtained for pUC-STS was found to match the published sequences of *sts *from peanut[21,22], but with several nucleotide changes. These changes were conserved in three clones sequenced, suggesting that the observed differences are the result of strain variation. These nucleotide changes caused a few silent mutations and nine changes in amino acid sequence (G6E, V31I, G46S, N202S, N234K, Q245K, G281D, K325N and T356E).

**Figure 2:**
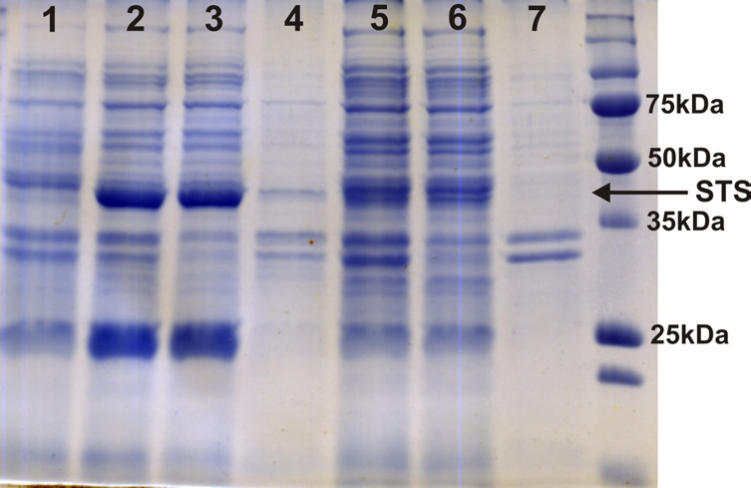
**Expression of *Arachis hypogaea *STS in *E. coli***. SDS-PAGE gel (12%) showing the overexpressed STS from peanut roots. The *sts *gene was cloned into the plasmid pUCMod as described in Materials and Methods. *E. coli *expressing STS, and a control culture with empty plasmid, were grown overnight in modified M9 medium at 30°C containing glycerol (lanes 1–4) or glucose (lanes 5–7). Cell lysates were prepared as described in Material and Methods. Samples are as follows: Lane 1: empty pUC control, Lane 2: pUC-STS grown in glycerol crude cell lysate, Lane 3: pUC-STS grown in glycerol soluble protein fraction, Lane 4: pUC-STS grown in glycerol insoluble protein fraction, Lane 5: pUC-STS grown in glucose crude cell lysate, Lane 6: pUC-STS grown in glucose soluble protein fraction and Lane 7: pUC-STS grown in glucose insoluble protein fraction, Lane 8: molecular weight standard.

Expression of recombinant STS was determined by SDS-PAGE, and the protein showed the expected molecular weight of near 42 kDa (Fig. 2, lane 2). When total cellular protein was separated into soluble and insoluble fractions by high-speed centrifugation, STS appeared to be almost entirely in the soluble fraction of the *E. coli *cell lysate, with a small amount found in the insoluble fraction (Fig. 2, lanes 3–4). The high level of expressed soluble STS is surprising as many plant derived genes are not well expressed in *E. coli *without codon optimization to remove rare or unusual plant codons. A reduced growth temperature of 30°C may have increased the solubility of the recombinant protein[23]. Previous work on flavonoid biosynthesis in *E. coli *also revealed a significant decrease in product titers when cells were cultivated in media containing glucose due to decreased enzyme expression levels (K. Watts, unpublished data). To investigate whether this phenomenon was also important for recombinant stilbene biosynthesis, we cultivated *E. coli *pUC-STS in modified M9 containing glucose instead of glycerol. Expression of STS in glucose containing media was significantly lower (Fig. 2, lane 5–7), and was found exclusively in the soluble protein fraction (Fig. 2, lane 6).

### Biosynthesis of resveratrol in *E. coli*

**Figure 3:**
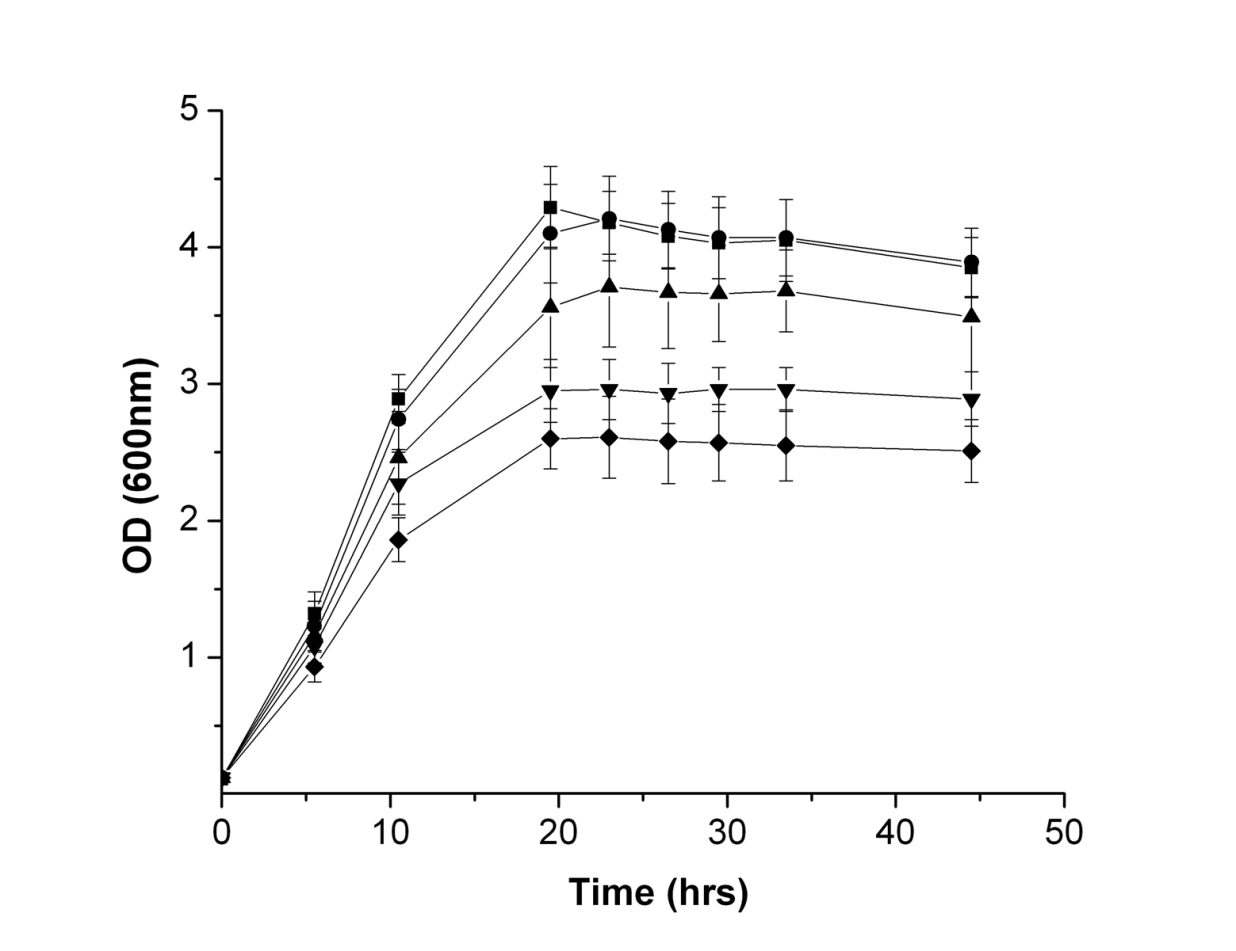
**Effect of increasing 4-coumaric acid concentrations on *E. coli *growth**. *E. coli *was grown in modified M9 medium containing glycerol with increasing concentrations of 4-coumaric acid to determine growth inhibition. Concentrations of added 4-coumaric acid were as follows: (●) 0 mM, (■) 2 mM, (▲) 6 mM, (▼) 12 mM and (◆) 20 mM. Growth was determined by OD measurement at 600 nm. Cultures were supplemented with varying concentrations of 4-coumaric acid once the starter culture reached an OD of 0.1. Data points represent the means of three independent measurements.

During our previous studies on flavonoid biosynthesis in *E. coli*, we observed that *E. coli *expressing a partial flavonoid pathway, consisting of 4CL1 and CHS, was able to take up exogenous 4-coumaric acid and synthesize naringenin[15]. To identify conditions for the biotransformation of phenylpropionic acids to stilbenes in an analogous reaction with 4CL1 and STS, *E. coli *was grown in the presence of increasing concentrations of 4-coumaric acid. Strain BW27784 with empty plasmids (pACMod + pUCMod) was used to mimic the culture conditions to be used later for stilbene biosynthesis. As shown in Fig. 3, no apparent growth inhibition was observed at 2 mM of added 4-coumaric acid as the growth curve closely follows that of the control culture without 4-coumaric acid at both 24 and 48 hrs of growth. However, inhibition of growth was observed at 6 mM, and at 20 mM of added 4-coumaric acid, the optical density (OD, λ = 600 nm) is reduced by approximately 38% after 24 hrs and 35% after 48 hrs. This effect is most likely due to 4-coumaric acid itself, rather than a drop in culture pH caused by the added phenylpropionic acid, as the pH of the cultures after 48 hrs did not change more than 1.0 pH unit compared to the control culture. Concentrations of 4-coumaric acid higher than 20 mM could not be easily reproduced due to precipitation of substrate in the culture media and on solid surfaces. Based upon these results, 1 mM was chosen as the phenylpropionic acid concentration for all subsequent biotransformation experiments.

**Figure 4:**
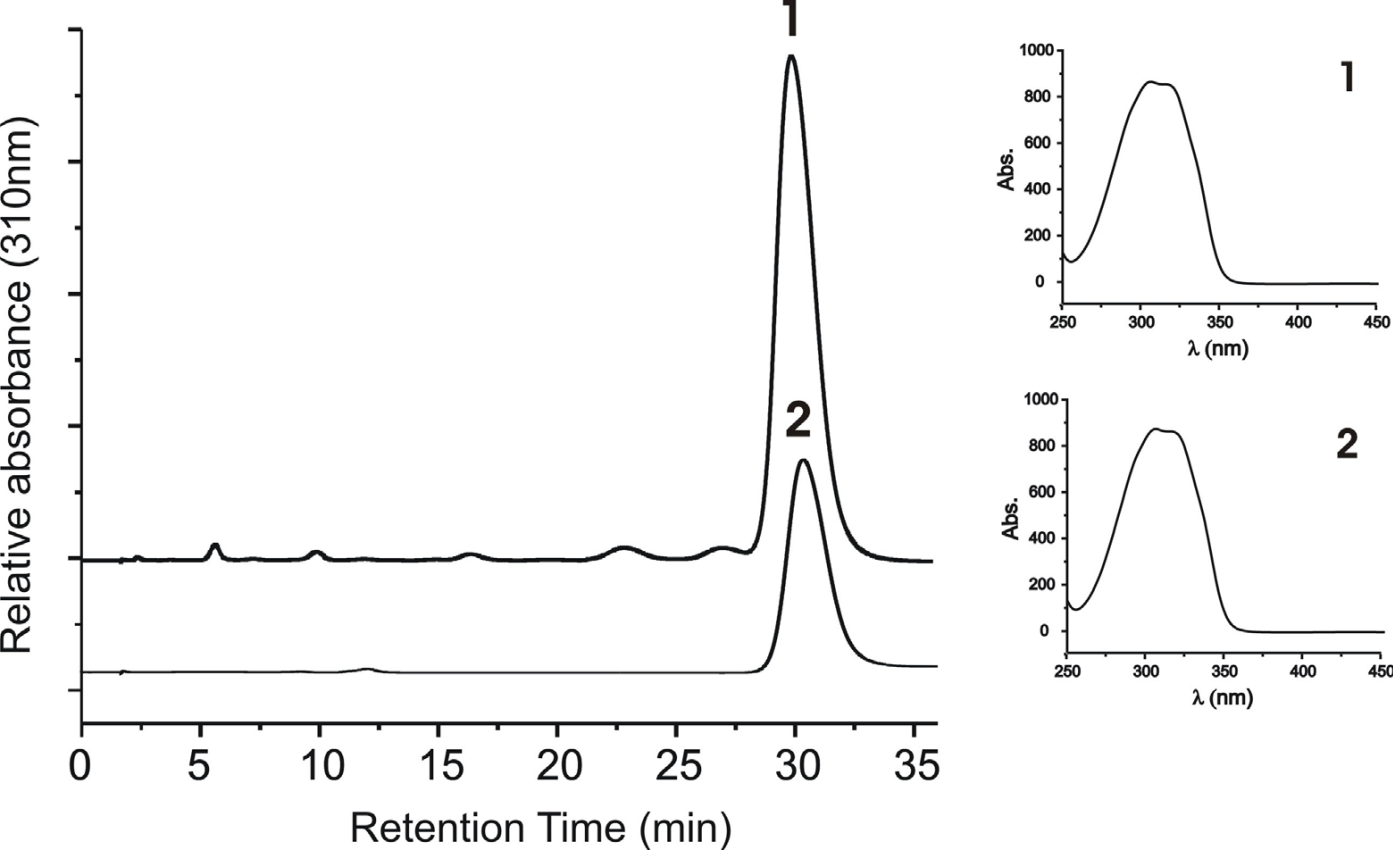
**HPLC analysis of resveratrol**. *E. coli *expressing 4CL1 and STS was cultured in modified M9 medium with glycerol, and containing 1 mM 4-coumaric acid, at 30°C for 24 hrs prior to extraction. Upper chromatogram shows extracted *E. coli *culture and bottom chromatogram shows resveratrol standard run under identical HPLC conditions. At right are the UV spectra for both recombinant resveratrol (1) (*m/z *227.1) and resveratrol standard (2) (*m/z *227.1). No residual 4-coumaric acid was detected by HPLC or LC-MS.

To examine the function of the peanut STS in *E. coli*, pUC-STS was transformed, along with pAC-4CL1, into *E. coli *strain BW27784 and grown in modified M9 media with glycerol. When the culture had grown to an OD of 0.1, 1 mM 4-coumaric acid was added. After 24 hrs growth at 30°C, the culture was extracted and analyzed for the expected stilbene compound resveratrol. The HPLC profile of the extract showed a peak with a retention time and UV spectrum identical to authentic resveratrol (Fig. 4). Control cultures of *E. coli *cells containing only pAC-4CL1 without STS, and supplemented with 1 mM 4-coumaric acid, did not show the same peak. Similarly, no resveratrol peak was detected in cultures of *E. coli *pAC-4CL1 + pUC-STS without added 4-coumaric acid. Liquid chromatography-mass spectrometry (LC-MS) analysis of this new compound showed a parent ion (M-H^+^) mass matching an authentic resveratrol standard (*m/z *227.1). This result demonstrates the first production of a stilbene compound in a bacterial host. Flavonoid biosynthesis has previously been reported in *E. coli*[15,24], but engineered stilbene biosynthesis has not been reported in this organism. This work therefore extends the range of plant polyketides that can be produced in *E. coli *to include stilbenes. The production of resveratrol in *E. coli *also opens up the possibility of engineering pathways for modified stilbene biosynthesis. Rational extension of the pathway with additional enzymes for methylation, glycosylation or prenylation will greatly expand the range of compounds that can be produced.

### Kinetics of resveratrol biosynthesis

**Figure 5:**
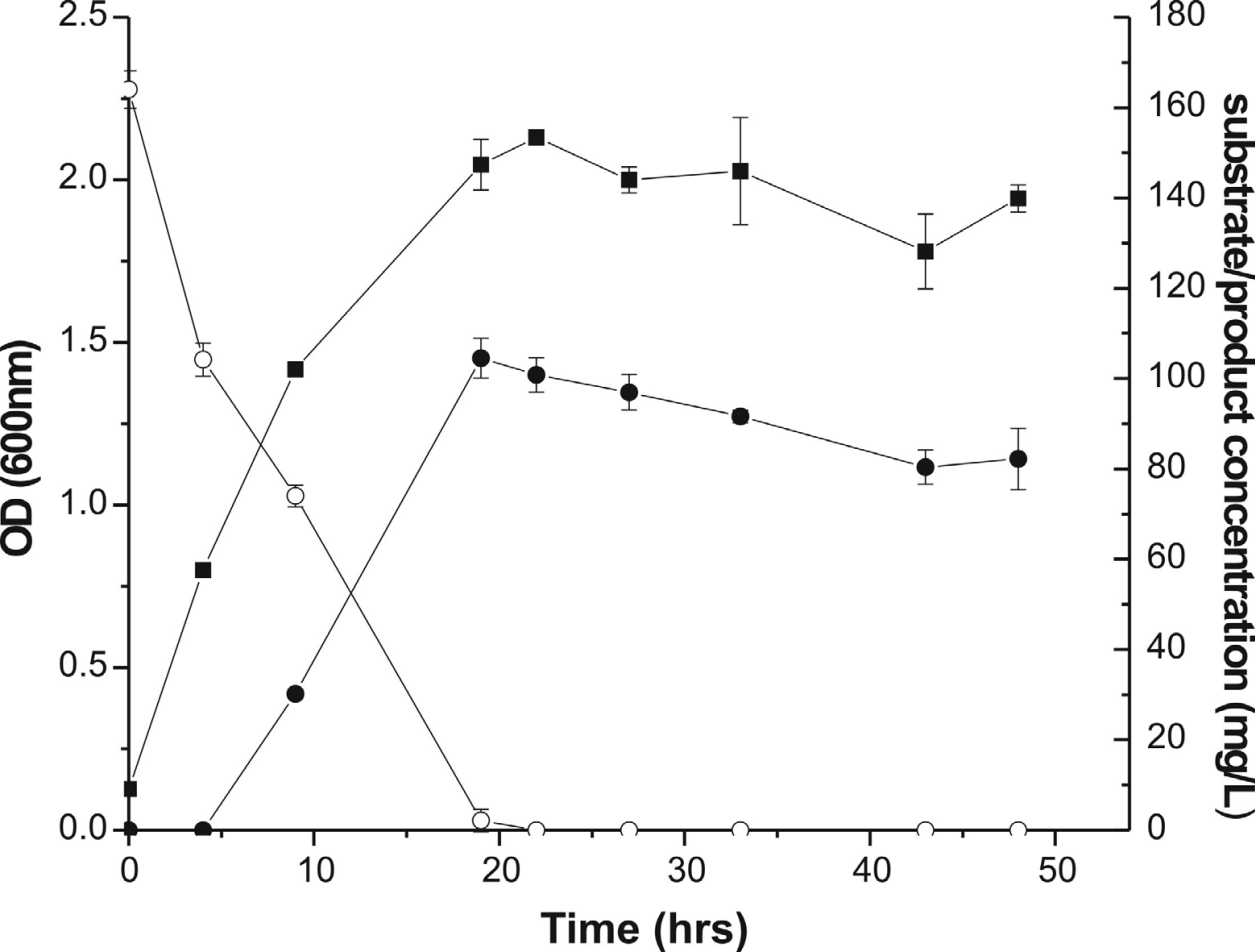
**Growth and production curves for resveratrol biosynthesis**. Culture of *E. coli *pAC-4CL1 + pUC-STS was supplemented with 1 mM 4-coumaric acid and grown for approximately 48 hours in modified M9 medium with glycerol at 30°C. Samples were removed periodically for extraction to determine the consumption of the substrate 4-coumaric acid (○) and the formation of the product resveratrol (●). Growth (■) was monitored spectrophotometrically by measuring OD at 600 nm. Data points represent the mean of three independent measurements.

*E. coli *pAC-4CL1 + pUC-STS was grown in shake flasks to determine growth and stilbene biosynthesis over time. Cultures were grown in modified M9 with glycerol to an OD of 0.1 when 1 mM 4-coumaric acid was added. Growth and product formation were subsequently followed for an additional 48 hours at 30°C. Resveratrol biosynthesis began almost immediately after addition of 4-coumaric acid and proceeded very quickly until all 4-coumaric acid present in the culture was consumed after approximately 20 hrs (Fig. 5). After 19 hrs of growth, 104.5 ± 4.4 mg/L resveratrol was detected in the culture media along with less than 2 mg/L 4-coumaric acid (Fig. 5). While 4-coumaric acid appeared to be completely consumed in the reaction after 22 hrs, the maximum amount of resveratrol detected corresponds to 460 μM, equal to a 46% conversion yield (mol/mol). Resveratrol levels decreased after reaching the maximum of 104.5 ± 4.4 mg/L at 19 hrs to 82.2 ± 6.8 mg/L after 48 hours, representing a 22% loss of extractable product over the course of 19 hours. The fate of the remainder of the 4-coumaric acid and the loss of extractable resveratrol are currently under investigation.

When this same experiment was repeated with modified M9 medium containing glucose, resveratrol production was substantially decreased. Resveratrol levels again reached a maximum value after approximately 20 hrs, but the amount was reduced to 3.8 ± 0.6 mg/L compared to 104.5 ± 4.4 mg/L obtained with glycerol. This drop in resveratrol production is likely a consequence of reduced STS expression in glucose containing M9 medium (Fig. 2, lanes 5–7), but could also be due in part to a transport phenomenon. Phenylpropionic acids are known to induce expression of enzymes responsible for transport and catabolism (*hca *and *mhp *operons) of these compounds in *E. coli*[25,26]. Additionally, expression of a regulatory protein (HcaR) controlling hydroxycinnamate transport and catabolism (*hca *operon) is subject to catabolite repression[25], suggesting that transport of phenylpropanoids into *E. coli *during growth in media containing glucose would be inhibited. This possibility is currently under investigation.

### Probing the biotransformation of diverse phenylpropionic acids by STS

**Figure 6:**
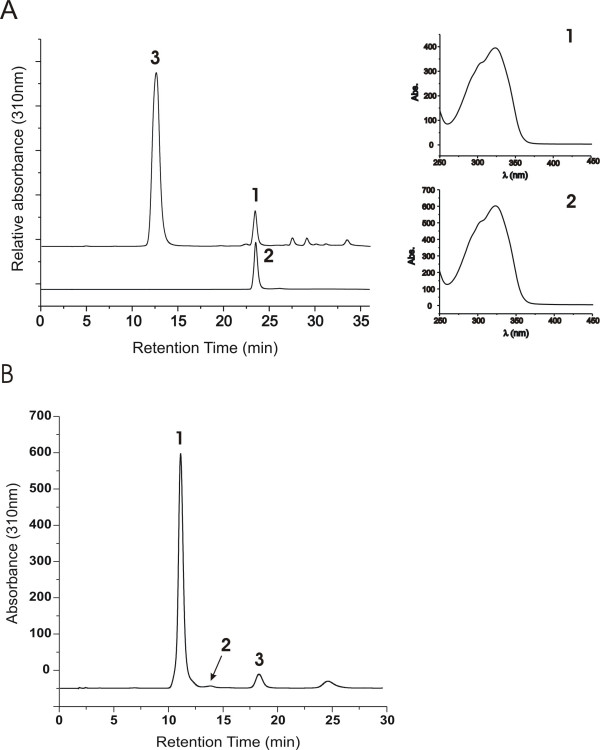
**HPLC analysis of cultures supplemented with caffeic acid or ferulic acid**. *E. coli *expressing 4CL1 and STS was supplemented with 1 mM substrate and grown in modified M9 medium at 30°C for 24 hrs prior to extraction. **Panel A**: Production of piceatannol from caffeic acid. Upper chromatogram shows extracted *E. coli *culture, bottom chromatogram shows piceatannol standard run under identical HPLC conditions. At right are the UV spectra for both recombinant piceatannol (1) (*m/z *243.1) and piceatannol standard (2) (*m/z *243.1). Peak 3 is residual caffeic acid (*m/z *179.2). **Panel B**: *E. coli *expressing 4CL1 and STS supplemented with ferulic acid. Peak 1 corresponds to residual ferulic acid (*m/z *193.1). Peak 2 corresponds to the ferulic acid derived tetraketide lactone (*m/z *301.1) and peak 3 is the corresponding triketide lactone (*m/z *259.1).

To determine the range of compounds that could be produced in recombinant *E. coli *co-expressing 4CL1 and STS, two additional phenylpropionic acid compounds were tested as substrates: caffeic acid or ferulic acid (see Fig. 1 for structures). When *E. coli *pAC-4CL1 + pUC-STS was grown in medium containing 1 mM caffeic acid, a new peak was detected in a HPLC chromatogram of the extract that was not present in an unsupplemented control culture. HPLC retention time, UV spectrum and parent ion mass (*m/z *243.1) of this new compound matched that of the stilbene piceatannol (Fig. 6A). Detectable levels of piceatannol reached 13.3 ± 0.3 mg/L after 24 hrs and 13.2 ± 0.6 mg/L after 48 hrs of growth.

This result marks the first production of a dihydroxylated stilbene in a recombinant host. The production of a stilbene from a dihydroxylated precursor (caffeic acid) is an important step in the further development of *E. coli *for stilbene biosynthesis as it circumvents the presumed cytochrome P450 monooxygenase mediated hydroxylation of resveratrol. While the enzyme catalyzing this reaction has not been described in plants, in humans the reaction is catalyzed by a cytochrome P450, CYP1B1[27]. Cytochrome P450 enzymes have traditionally been difficult to functionally express in *E. coli *due to a lack of a compatible P450 reductase[28]. While functional plant P450 expression in *E. coli *may ultimately be accomplished, as it has in yeast[29], it will most likely require extensive protein engineering efforts, such as translational fusions to compatible reductases[30]. This finding also agrees with what has been seen *in vitro *with peanut STS and this substrate. While 4-coumaric acid was reported to be the preferred substrate for this enzyme (Km = 2 μM), caffeic acid was also utilized to a small extent[31]. The relative amount of piceatannol produced *in vitro *from caffeoyl-CoA was approximately 12 fold lower than resveratrol produced from an equivalent amount of 4-coumaroyl-CoA[31], and this is in close proximity to what is seen *in vivo *with our *E. coli *system.

The bulkier phenylpropionic acid substrate ferulic acid, which has one of the two hydroxyl groups present in caffeic acid methylated, did not yield the corresponding fully cyclized stilbene compound isorhapontigenin. Instead, extracts from *E. coli *pAC-4CL1 + pUC-STS cultures supplemented with ferulic acid yielded two new peaks on HPLC which were identified by mass spectrometry as the corresponding triketide (*m/z *259.1) and tetraketide (*m/z *301.1) lactone intermediates (Fig. 6B). The major peak observed in the ferulic acid supplemented culture extract, however, always corresponded to unconverted ferulic acid, indicating that either substrate utilization by 4CL1 in *E. coli *may be limiting or that the reaction of STS with feruloyl-CoA is inefficient. *In vitro*, feruloyl-CoA was reported to be converted to the corresponding stilbene, albeit at low levels[31]. This suggested that the concentration of feruloyl-CoA was limiting in *E. coli *due to the CoA ligase 4CL1.

### Substitution of 4CL1 with the feruloyl-CoA ligase 4CL4

Ferulic acid is known to be a poor substrate for the 4CL1 enzyme from *A. thaliana *used in our studies[32]. However, a new 4CL1 homolog, 4CL4 from *A. thaliana*, was recently shown to preferentially use ferulic acid and sinapic acid as substrates[33]. Therefore, to investigate whether the substrate specificity of 4CL1 was a limiting step in stilbene biosynthesis from ferulic acid, 4CL4 was cloned and co-expressed with STS. When *E. coli *pAC-4CL4 + pUC-STS was grown in the presence of 1 mM ferulic acid, no detectable isorhapontigenin was found by HPLC or LC-MS analysis (data not shown). As with previous cultures expressing 4CL1 and STS, similar amounts of triketide and tetraketide lactones, and unconsumed ferulic acid, were detected. The presence of high levels of residual ferulic acid could indicate that the reaction of STS with feruloyl-CoA is slow, causing an accumulation of feruloyl-CoA, which can be converted back to ferulic acid by the action of a soluble thioesterase, as has been observed during attempts to purify aromatic CoA thioesters from *E. coli*[34].

The result with 4CL4, along with the previous result with 4CL1, suggests that STS can use ferulic acid as a starter unit *in vivo*, but it is unable to properly extend and fold the intermediates formed, resulting in lactone derailment products. These triketide and tetraketide lactones are typically found with unnatural substrates in CHS and STS *in vitro *assays. The presence of lactone derailment products demonstrates the ability of these enzymes to accept a wide range of starter units, but due to limitations in the size of the active site, they are not properly extended and/or folded, and the resulting intermediates are released[35]. We have observed *in vivo *that CHS can also use ferulic acid as a starter unit, and like STS, it does not properly fold the intermediates, producing only the lactone derailment products. Mutants of CHS designed to expand the active site cavity, expressed together with 4CL4 in *E. coli*, produce differing ratios of triketide and tetraketide lactones *in vivo *(K. Watts, unpublished data). This suggests that expansion of the STS active site cavity may similarly lead to mutants that can more readily accommodate the larger size of ferulic acid derived polyketide intermediates, and eventually produce the properly folded stilbene structure.

## Conclusion

For the first time, biosynthesis of stilbene compounds by engineered *E. coli *was demonstrated. The medicinally important compound resveratrol was produced at a level of over 100 mg/L in about 20 hrs of growth, at which time 4-coumaric acid was no longer detectable. This is a significantly higher yield of resveratrol than the 1–2 μg/L levels previously reported for engineered *Saccharomyces cerevisiae*[36]. Analysis of protein expression data suggests that the high level of soluble STS appears to be one likely reason for this observation. The quantity of the stilbene piceatannol produced from caffeic acid was also relatively high, around 13 mg/L. While the amount of resveratrol produced is quite high, the conversion yield is less than 50% from the added substrate 4-coumaric acid. This may be partially explained by the capability of *E. coli *to degrade aromatic acids, including phenylpropanoids[37]. Efforts are currently underway to elucidate the mechanisms of substrate disappearance in order to gain insight into phenylpropanoid transport and metabolism in *E. coli*.

Ferulic acid was not converted to the corresponding stilbene structure, isorhapontigenin, by *E. coli *expressing 4CL1, or a ferulic acid specific CoA ligase, 4CL4, in conjunction with STS. The use of 4CL4 was meant to overcome the poor utilization of ferulic acid by 4CL1, but use of 4CL4 had no apparent effect on product formation. It would appear then that feruloyl-CoA utilization by STS is the limiting step in the pathway. CHS likewise does not produce a flavanone product from ferulic acid, but produces tri- and tetraketide lactones. Restricting the active site cavity of CHS with this substrate produces increased triketide lactone[38], suggesting that expansion of the active site may have the opposite effect. Indeed, simple mutations in CHS, and closely related enzymes, have resulted in dramatic changes in substrate specificity and product formation[35,39,40]. Since STS has been postulated to have evolved from CHS[41], the ability to expand and modify the active site of CHS may as well be possible in STS.

This work opens up alternative routes for the production of additional stilbene structures from phenylpropionic acid precursors using recombinant *E. coli *cells. Through manipulation of the biosynthetic enzymes controlling product formation, and eliminating unwanted reactions within the host, improvements may be seen that result in efficient utilization of an expanded range of substrates.

## Methods

### Chemicals

Caffeic acid, ferulic acid and piceatannol were purchased from Sigma Aldrich (St. Louis, MO). 4-coumaric acid was purchased from ICN (Aurora, OH) and resveratrol was from Calbiochem (San Diego, CA). All solvents used were of HPLC grade and purchased from Fisher Scientific (Pittsburgh, PA). HPLC grade water was purchased from Mallinckrodt Chemicals (Phillipsburg, NJ). T4 DNA ligase and Vent DNA polymerase were from New England Biolabs (NEB, Boston, MA). Restriction enzymes were from NEB or Promega (Madison, WI) and restriction enzyme buffers were the SuRE/Cut buffers from Roche (Indianapolis, IN).

### Strains and culture conditions

All cloning and DNA manipulations were carried out in *E. coli *strain JM109 by following standard techniques described elsewhere[42]. After DNA sequencing, plasmids were transformed into *E. coli *strain BW27784[43] (*E. coli *Genetic Stock Center, New Haven, CT) for stilbene biosynthesis. *E. coli *cultures were grown at 30°C with 250 RPM shaking in a modified M9 or Luria-Bertani (LB) medium, supplemented with carbenicillin or ampicillin (100 mg/mL), and chloramphenicol (50 mg/mL), if necessary. M9 medium was modified by addition of yeast extract (1.25 g/L) and glycerol (0.5% v/v) or glucose (1% v/v) into standard M9 medium [42].

### Plant growth and cDNA preparation

*A. hypogaea *(peanut) seeds were purchased from Burpee Seed Company (Warminster, PA). Plants were grown on a well-lit window ledge, at approximately 23°C, for two weeks prior to harvesting. Tissue samples were cut and frozen immediately in liquid nitrogen and stored at -80°C prior to use. mRNA was extracted using the Qiagen RNeasy Plant Mini Kit (Valencia, CA), followed by DNase I digestion with the DNA-free Kit from Ambion (Austin, TX). mRNA was annealed to oligo dT (Invitrogen, Carlsbad, CA) during a single 65°C treatment for 5 minutes, put on ice, and followed by RT-PCR using Transcriptor Reverse Transcriptase from Roche. Following the RT-PCR step, cDNA was used immediately for subsequent PCR.

### Cloning and pathway assembly

Stilbene synthase (STS) was cloned from freshly prepared root cDNA with gene specific primers designed from the published sequence [22] [Genbank: AB027606]. Primers included an *Xba*I site, followed by an optimized Shine-Dalgarno sequence (5'*-TCTAGA*AGGAGGATTACAAA**ATG**3') and the start codon, with 10–15 additional nucleotides from the gene sequence immediately after the start codon. Reverse primers contained a *Not*I site for directional cloning into pUCMod, a modified pUC19 plasmid with a deleted operator sequence for constitutive expression from the *lac *promoter [20]. PCR was carried out with Vent DNA polymerase and conditions were as follows: 94°C for 2 minutes, 30 cycles of 94°C for 30 seconds, 50°C for 30 seconds, 72°C for 1 minute followed by a final extension step at 72°C for 4 minutes. The resulting STS PCR product was cloned into the *Xba*I and *Not*I sites of pUCMod to create pUC-STS.

4CL4 was cloned from a commercial *A. thaliana *cDNA library with primers designed from the published sequence [33] [Genbank: AY376731]. Primers were designed the same as above, with a 5' *Xba*I site and a 3' *Not*I site for directional cloning into pUCMod to create pUC-4CL4. PCR conditions were the same as described above. 4CL4 was subcloned, along with the constitutive *lac *promoter from pUC-4CL4, into the *Bam*HI site of pACMod to create pAC-4CL4. 4CL1 was subcloned from pBAD-4CL [15] into the *Nco*I site of pUCMod with gene specific primers containing *Nco*I sites in both the forward and reverse direction to create pUC-4CL1, which is also transcribed from the constitutive *lac *promoter. PCR conditions were the same as above. 4CL1 was later subcloned to the *Bam*HI site of pACMod to create pAC-4CL1.

### Protein expression analysis

*E. coli *BW27784 was transformed with pUC-STS and grown overnight at 30°C in 5 mL of modified M9 media containing glycerol or glucose. This culture was used for 1:100 inoculation into 50 mL modified M9 containing glycerol or glucose and grown at 30°C for an additional 24 hours. Cells were harvested by centrifugation at 4000 × g, washed with 10 mL of phosphate buffer (50 mM NaPO_4_, pH 8.0) and the OD was determined at 600 nm. Cells were diluted to equivalent ODs (1.0) with phosphate buffer and centrifuged. Cell pellets were resuspended in 10 mL phosphate buffer and lysed by sonication (Branson, Danbury, CT). Lysate was centrifuged at 10,000 × g for 30 minutes to pellet insoluble material. Following centrifugation, cleared lysate (10 mL) was transferred to a fresh conical tube, and pelleted material was resuspended in 10 mL fresh phosphate buffer. Equal volumes (50 μL) from each fraction (crude, soluble and insoluble) were removed and mixed with 50 μL SDS running buffer and boiled briefly at 100°C. For gel analysis, 10 μL of each sample mixture was loaded and run on a 12% gel.

### Substrate inhibition curves

For determination of growth inhibition by 4-coumaric acid, 500 mL of *E. coli *BW27784 pACMod + pUCMod was grown at 30°C to an OD of 0.1 – 0.2 and split into 5 flasks containing 50 mL modified M9 medium with glycerol. Growth media was supplemented with 0, 2, 6, 12 or 20 mM 4-coumaric acid in 200 μl DMSO. Cultures were grown for an additional 48 hrs at 30°C, and this process was repeated for three independent measurements. 1 mL samples were removed periodically to record OD at 600 nm.

### Biotransformation

5 mL overnight cultures of *E. coli *pAC-4CL1 or pAC-4CL4 + pUCMod or pUC-STS were inoculated 1:100 into 50 mL modified M9 medium with glycerol containing chloramphenicol and carbenicillin. For biotransformation experiments, 1 mM 4-coumaric acid, caffeic acid or ferulic acid in 200 μl DMSO was added to *E. coli *cultures at an initial OD of 0.1-0.2. Cultures grew for an additional 48 hrs at 30°C prior to harvest and extraction.

### Growth and production curves

Overnight culture of *E. coli *pAC-4CL1 + pUC-STS was inoculated 1:200 into 700 mL fresh modified M9 medium containing glycerol or glucose, and supplemented with chloramphenicol and carbenicillin. The culture was grown to an OD of 0.1 – 0.2, split into three separate 500 mL flasks, each containing 200 mL of culture, and supplemented with 1 mM 4-coumaric acid. Growth was continued for an additional 48 hrs at 30°C and OD was monitored at 600 nm. 1 mL samples were removed periodically for analysis and quantification of 4-coumaric acid and reaction products.

### Extraction of culture media

Previous work had shown that less than 5% of products and phenylpropionic acids were found in the cell pellets [15], therefore only culture media was extracted. For extraction, 1 mL of the culture was centrifuged at maximum speed (>13,000 RPM) to pellet cells. Media was decanted to a fresh 1.5 mL microfuge tube and the pH was adjusted by addition of 50 μl hydrochloric acid (1N), followed by overnight freezing at -20°C. Tubes were thawed at room temperature and extracted twice with an equal volume (1 mL) of ethyl acetate. Ethyl acetate was dried under nitrogen gas, and the dried residue was resuspended in 100 μL methanol. All samples were stored at -20°C prior to HPLC and LC-MS analysis.

### HPLC analysis

10 μl of extract was applied to a Zorbax RX-C18 column (4.6 × 150 mm, 5 μm; Agilent Technologies, Palo Alto, CA) using an Agilent 1100 HPLC system equipped with a photodiode array detector. Resveratrol and ferulic acid derived products were eluted with an isocratic mobile phase of water containing 0.1% trifluoroacetic acid (A) and methanol containing 0.1% trifluoroacetic acid (B) in a ratio of 73:27 (A:B, v/v) with a flow rate of 1.0 mL/min. Piceatannol was eluted with a flow rate of 0.5 mL/min using the following conditions: from 0–10 min 75:25 A:B, followed by a gradient from 75:25 A:B to 50:50 A:B in 15 minutes, followed by 5 min 50:50 A:B. Compound peaks were identified by comparison to retention times and UV/Vis spectra of standard compounds and mass spectrometry. For quantification of products, standard curves were constructed by plotting peak areas of known quantities of stilbene standards.

### LC/ESI-MS analysis

LC-Mass spectrometry was carried out with a LCQ mass spectrophotometer (Thermo Finnigan, USA) equipped with a Zorbax RX-C18 column (4.6 × 250 mm, 5 μm; Agilent) and eluted at 1.0 mL/min under isocratic conditions of water:methanol (70:30). Mass fragmentation spectra of standard compounds and the extracted compounds were monitored in a mass range of *m/z *100–500 with a negative electron spray ionization (ESI) interface as described previously [15]. Negative ion values for standard compounds were as follows: 4-coumaric acid (*m/z *163.1), caffeic acid (*m/z *179.1), ferulic acid (*m/z *193.1), resveratrol (*m/z *227.1) and piceatannol (*m/z *243.1).

## Authors' contributions

KTW designed experiments and carried out all cloning, protein expression, analytical studies and wrote the manuscript. PCL performed mass spectrometry and contributed to experimental design. CSD contributed to experimental design and wrote the manuscript. All authors reviewed and approved the final manuscript.

## Supplementary Material

Additional file 1 **Amplification of *sts *from *A. hypogaea *root cDNA**. Watts, et al. Supplemental figure 1. cDNA was isolated from *A. hypogaea *and probed with *sts *specific primers. The cDNA was prepared from roots and root hairs together (lanes 1 and 2) and fully opened leaves (lanes 4 and 5). Lane 3 is a 1 kilobase DNA ladder. For PCR, either 0.5 μl (lanes 1 and 4) or 2 μl (lanes 2 and 5) of freshly prepared cDNA was used in a 100 μl PCR reaction. The obtained PCR product runs at approximately 1200 bp on a 1% agarose gel, which closely matches the expected size of 1170 bp for *sts *from *A. hypogaea*. Click here for file
