# Supplementary figures and images for: Biosynthesis of plant-specific stilbene polyketides in metabolically engineered Escherichia coli
Source: BMC Biotechnol. 2006 Mar 21;6:22. doi: 10.1186/1472-6750-6-22 (PMC1435877; doi:10.1186/1472-6750-6-22)

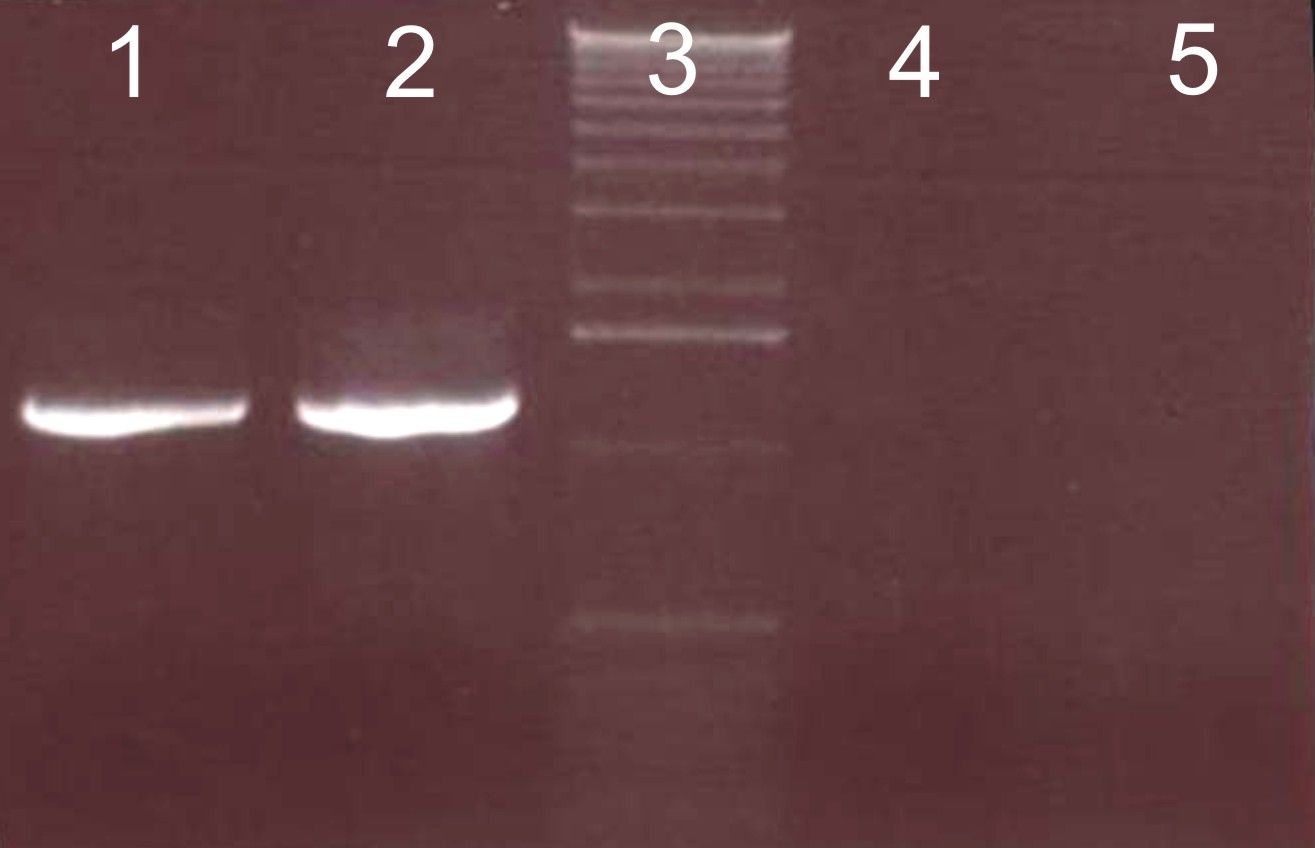

Supplement: Additional file 1 — Amplification of sts from A. hypogaea root cDNA. Watts, et al. Supplemental figure 1. cDNA was isolated from A. hypogaea and probed with sts specific primers. The cDNA was prepared from roots and root hairs together (lanes 1 and 2) and fully opened leaves (lanes 4 and 5). Lane 3 is a 1 kilobase DNA ladder. For PCR, either 0.5 μl (lanes 1 and 4) or 2 μl (lanes 2 and 5) of freshly prepared cDNA was used in a 100 μl PCR reaction. The obtained PCR product runs at approximately 1200 bp on a 1% agarose gel, which closely matches the expected size of 1170 bp for sts from A. hypogaea. [file 1472-6750-6-22-S1.jpeg]
